# Rare Diagnostic and Clinical Manifestations in an Acute Hepatitis A Infection: A Case Report

**DOI:** 10.7759/cureus.29194

**Published:** 2022-09-15

**Authors:** Wesley D Figg, David L Crawford, Tristen N Taylor, Mayar Al Mohajer

**Affiliations:** 1 Department of Medicine, Baylor College of Medicine, Houston, USA

**Keywords:** rare entity, atypical presentation, extrahepatic manifestation, viral hepatitis a, delayed diagnosis

## Abstract

The hepatitis A virus (HAV) is a common cause of infectious hepatitis worldwide. In adults, clinical manifestations typically involve fever, nausea/vomiting, fatigue, abdominal pain, and jaundice, although rarer manifestations may be observed. Acute hepatitis A infection is detected via anti-HAV IgM antibodies, which are present in almost all patients at symptom onset. In this case, we present a patient who not only tested negative for acute HAV infection at symptom onset, but also presented with uncommon, extrahepatic manifestations including maculopapular skin rash and polyarthralgia. Wariness of such a presentation can facilitate the timely diagnosis of atypical cases of HAV infection.

We report the case of a 51-year-old man who presented with fever, abdominal pain, headaches, and diarrhea for one week with elevated liver enzymes and leukocytosis. Workup consisting of viral hepatitis panels, various infectious studies, and rheumatologic antibody titers did not initially reveal an etiology for the patient’s presentation. Computed tomography (CT) abdomen and pelvis, abdominal ultrasound, magnetic resonance cholangiopancreatography (MRCP), and hepatobiliary iminodiacetic acid (HIDA) scan did not reveal acute pathology. The patient’s symptoms worsened over the following days, and he additionally developed bilateral wrist pain, digital arthralgias, paraspinal back pain, diffuse muscular weakness, and a pruritic maculopapular rash affecting the flanks and extremities. Eventually, viral hepatitis studies were repeated which revealed elevated levels of anti-HAV IgM antibodies, indicating acute hepatitis A infection. The patient was treated supportively while hospitalized with subsequent improvement of symptoms and lab abnormalities. Since discharge, the patient had not experienced persistent sequelae of the disease.

This case of acute viral hepatitis A infection is notable for two reasons: (1) the patient experienced uncommon, delayed, extrahepatic manifestations of disease, and (2) the initial viral hepatitis studies revealed undetectable anti-HAV IgM levels despite having experienced symptoms of illness for several days. This case suggests that repeat viral hepatitis testing may be warranted in patients who continue to experience manifestations of the infection after initially testing negative. It also emphasizes the importance of recognizing potential atypical manifestations of acute hepatitis A infection.

## Introduction

Hepatitis A virus (HAV), a nonenveloped, single-stranded RNA virus of the *Picornaviridae *family, is a virus that causes acute infectious hepatitis [1]. Hepatitis A, which is typically spread via the fecal-oral route, is endemic in developing countries due to factors such as poor sanitation or contamination of food or water. In contrast, since the implementation of the hepatitis A vaccine and improved sanitation, infection rates in developed countries have substantially declined making acute hepatitis A infection relatively uncommon [1]. The average incubation period of hepatitis A is roughly 30 days, and the manifestations of acute infection are age-dependent [2].

The typical clinical manifestations of acute hepatitis A virus infection vary depending on age. In children under the age of 6 years old, roughly 70% of acute infections are asymptomatic, whereas about 70% of adult patients experience jaundice and markedly elevated aminotransferases [3]. Adult hepatitis A infection most commonly presents with abrupt onset of fever, malaise, nausea or vomiting, abdominal pain, dark-colored urine, and jaundice [4]. Atypical manifestations of acute HAV infection that have been reported primarily include cholestasis, relapsing and remitting infection, and autoimmune phenomena such as chronic autoimmune hepatitis [5]. However, symptoms such as a pruritic skin rash, diarrhea, and arthralgia have seldom been reported in the literature [4,6]. In one such case, a biopsy of the patient’s skin rash revealed leukocytoclastic vasculitis with direct immunofluorescence studies showing vascular deposition of IgM and C3 complement [7]. Recognition of such variable manifestations of HAV infection in adults is critical for the timely and accurate diagnosis of HAV infection.

The symptoms of acute hepatitis A infection in adults are nonspecific, vary widely, and may include fever, nausea/vomiting, fatigue, abdominal pain, and/or jaundice [8]. IgM antibodies are present in almost all patients at the time of their initial presentation and detection of these antibodies is the gold-standard method for diagnosis [9]. The methods for detecting these antibodies are highly sensitive and specific, thus the probability of obtaining a falsely negative test result at the onset of clinical symptoms is exceedingly low.

In this case, however, we report a patient who presented several days after the onset of symptoms and initially tested negative for acute hepatitis A infection, but eventually tested positive 16 days later. Additionally, this patient exhibited a widespread, maculopapular rash and polyarthralgia - symptoms that are rarely associated with acute hepatitis A infection. The uncommon diagnostic course and atypical manifestations presented in this case exemplify a unique presentation of acute hepatitis A infection.

This article was previously presented as a poster at the 2022 Baylor College of Medicine Department of Medicine Housestaff Research Symposium on April 11-15, 2022.

## Case presentation

A 51-year-old Caucasian man with hypertension living in the United States presented with fever, abdominal pain, headaches, and diarrhea for seven days. Initial laboratory tests revealed leukocytosis and mildly elevated liver enzymes. Computed tomography (CT) of the abdomen, magnetic resonance cholangiopancreatography (MRCP), abdominal ultrasound, and hepatobiliary iminodiacetic acid (HIDA) scan were performed and did not reveal acute pathology. Further workup included a viral hepatitis panel (eight days from symptom onset), autoimmune antibody titers, and anti-cytomegalovirus (CMV) IgG levels, all of which returned negative. Notably, the viral hepatitis panel did not reveal acute hepatitis B or C infection, and anti-HAV IgM and IgG antibodies were not detected. The patient was given empiric treatment with ceftriaxone for suspected cholangitis and was discharged home on hospital day 10.

For two days after discharge, the patient continued to experience daily fevers and developed new bilateral wrist pain and interphalangeal arthralgias, bilateral paraspinal back pain, and diffuse muscular weakness. His diarrhea and vomiting had resolved. The patient then presented to a different hospital (our facility) for re-evaluation at this time. The patient was febrile to 38.3°C (101.0°F) and a physical exam on this admission notably revealed proximal and distal interphalangeal arthralgias with active and passive range of motion on the third and fourth digits of both hands. The abdominal exam demonstrated a non-distended abdomen without hepatosplenomegaly, a lack of tenderness to palpation of all four abdominal quadrants, and normal bowel sounds. There was no evidence of jaundice or scleral icterus. The patient works as a salesman and reports no recent sexual activity, travel, drug use, seafood consumption, exposure to unclean water, or contacts with a known hepatitis A diagnosis. Notably, the patient reported not previously receiving vaccination against hepatitis A, serving as his only apparent risk factor for acquiring acute hepatitis A infection. Extensive infectious and rheumatologic workup ensued (Table 1) with all results returning negative and unrevealing for an etiology of the patient’s symptoms and lab abnormalities. Additionally, his liver function tests and alkaline phosphatase levels continued to rise throughout admission.

**Table 1:** Full rheumatologic and infectious workup CMV: Cytomegalovirus

Test
Anti-nuclear antibody
SS-A/Ro antibody
SS-B/La antibody
Sm (Smith) antibody
Smooth muscle (SM) antibody
Anti-nRNP/Sm antibody
Scl-70 (Scleroderma) antibody
Anti-mitochondrial antibody
Anti-double strand DNA antibody
Thyroid peroxidase antibodies
Anti-striated muscle antibody
Anti-parietal cell antibody
Complement C4
Hepatitis B surface antigen
Hepatitis B core IgM antibody
Hepatitis C antibody
CMV IgG antibody
Serum heterophile antibody titer by latex agglutination
HIV-1/2 antibody
HIV P24 antigen
Clostridium difficile toxin A and B
SARS-CoV-2 RNA PCR
HSV-1/2 DNA PCR
Actin (smooth muscle) IgG antibody
Mitochondria M2 IgG antibody
Rapid Plasma Reagin

**Figure 1:**
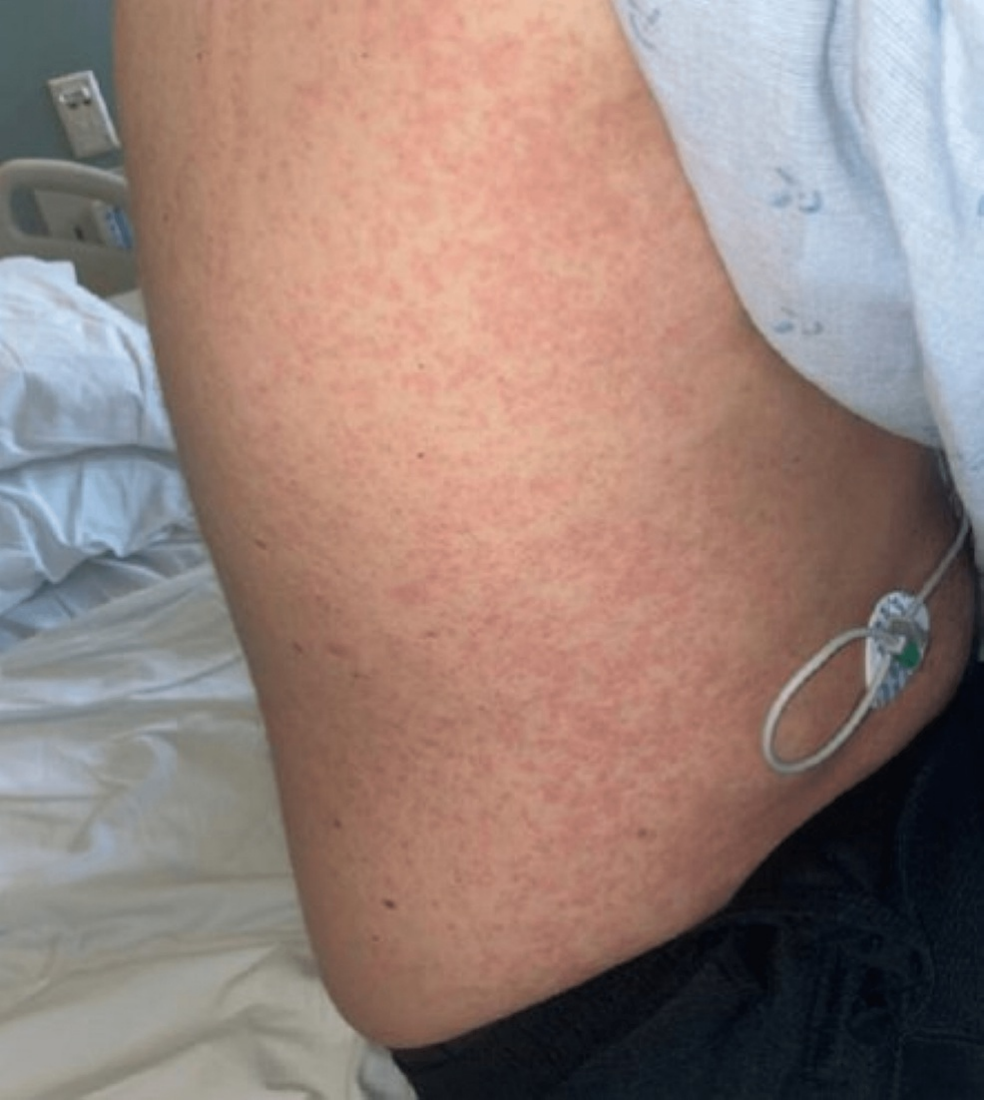
Rash affecting the right flank

Twenty-three days after the onset of symptoms (five days after presenting to our facility), the patient developed new-onset diffuse, pruritic maculopapular rash involving his bilateral flanks (Figure 1) and proximal upper and lower extremities. Additionally, the patient’s aspartate aminotransferase (AST), alanine aminotransferase (ALT), and alkaline phosphatase levels peaked at this time as well at 128 U/L, 154 U/L, and 159 U/L, respectively. He continued to experience polyarthralgia with a similar distribution as previously mentioned.

Given the lack of an explanation for the patient’s presentation, the decision to retest for viral hepatitis A, B, and C was made (24 days after symptom onset) which consequently revealed a positive anti-HAV IgM serology, indicating acute hepatitis A viral infection and providing a plausible unifying diagnosis (Table 2). The patient continued to receive supportive treatment and was given instructions to follow up with infectious disease specialists upon discharge. His symptoms improved over the course of one week and no persistent complications from the infection had become apparent since discharge.

**Table 2:** Timeline and salient features of illness course AST: Aspartate aminotransferase; ALT: Alanine aminotransferase

Days since symptom onset	Notable hospital course
0	Onset of fevers/chills, headache, abdominal pain, and diarrhea
7	Presents to first hospital; CT abdomen and right upper quadrant ultrasound negative
8	Initial viral hepatitis panel negative
9	Negative hepatobiliary iminodiacetic acid (HIDA) scan
13	Repeat CT abdomen negative
16	ALT 53 U/L, WBC 15.36 cells per cubic mm, alkaline phosphatase 121 U/L; discharged home with presumed cholangitis and empiric treatment with ceftriaxone
18	Presents to our facility with persistent fevers/chills, headaches, new bilateral hand pain, back pain, and bilateral digital arthralgias
20	Leukocytosis peaks at 16.8 cells per cubic mm
23	Develops new-onset maculopapular rash AST, ALT, alkaline phosphatase peak at 128 U/L, 154 U/L, and 159 U/L respectively
24	Repeat viral hepatitis panel reveals elevated anti-HAV IgM titer
25	Improvement of transaminitis and alkaline phosphatase levels, improvement of polyarthralgia

## Discussion

This case proved diagnostically challenging for several reasons. The patient initially tested negative for HAV after the onset of symptoms, he presented with symptoms that are uncommon for acute HAV infection, and since the development of the HAV vaccine, rates of infection have declined significantly in developed countries making HAV an often-overlooked disease. Interestingly, however, reported cases of hepatitis A infection in the United States have increased considerably since 2016 due to the occurrence of large person-to-person outbreaks [10]. Since the beginning of said outbreaks, 43,705 cases of acute hepatitis A infection have been reported across 37 states (as of February 11, 2022), resulting in 26,659 hospitalizations and 420 deaths [11]. The present case occurred in Texas and, according to the Texas Department of State Health Services, there has been a recent rise in the number of reported cases of hepatitis A infection [12]. Eighty-eight cases were reported in 2018, 160 cases reported in 2019, and 223 cases reported in 2020 [12]. This sudden change in the epidemiology of HAV infection highlights the importance of recognizing potential signs and symptoms of HAV infection, as well as understanding instances where retesting for HAV infection may be warranted.

Following exposure, the incubation period of HAV ranges from 20 to 45 days, and peak viremia is achieved near the time of onset of abnormal liver function tests [13]. Anti-HAV antibodies are almost uniformly present at the time of onset of clinical symptoms, and acute infection is diagnosed via the detection of anti-HAV IgM antibodies in the patient’s serum by highly sensitive and specific commercially available immunoassays [14]. In a study performed by Lee et al., 10.9% of patients that were later confirmed on repeat testing to have acute hepatitis A infection initially lacked anti-HAV IgM antibodies, as seen in the present case [15]. This study revealed that the patients who initially tested negative for anti-HAV IgM antibodies shared certain characteristics, such as higher rate of fever and lower aminotransferase and bilirubin levels compared to patients with an initially positive anti-HAV IgM serology, similar to our observations of the present case. Therefore, in the setting of clinical manifestations concerning for infectious hepatitis in which an alternative explanation has not been discovered, it is important to consider retesting for HAV after an initially negative test.

Risk factors for HAV infection include travel to endemic regions, intravenous drug use, multiple blood transfusions, male homosexual activity, and occupations such as childcare, sanitation, and healthcare [16,17]. Primary prevention of HAV includes avoidance of risk factors, active immunization, and passive immunization. In areas with high rates of hepatitis A, nearly all inhabitants have previously acquired asymptomatic HAV infection in childhood, providing future protection against clinical hepatitis A and negating the need for vaccination [16]. When HAV vaccination is indicated, such as when residing in regions with intermediate endemicity or when traveling to countries with a high incidence of hepatitis A, it is typically offered as a two-dose series beginning after 12 months of age [16,18]. Older children, teens, and adults who were not previously immunized may also receive the HAV vaccine [18]. People with preexisting chronic liver disease have a higher risk of developing fulminant hepatitis when infected with hepatitis A and thus should be routinely vaccinated for HAV [16]. Following exposure to HAV, vaccination is indicated in all individuals aged one year and older within two weeks of exposure. In addition, hepatitis A immune globulin (IG) may be co-administered based on age, immune status, comorbidities, or in place of vaccination in those for whom vaccines are contraindicated [19].

Currently, there are no drugs targeted for the treatment of acute hepatitis A infection; thus, individuals confirmed to have an infection are managed supportively and treated symptomatically with the use of antiemetics, intravenous fluids, and antipyretics as indicated [3,20]. Given that the identified risk factors for acquiring acute HAV infection in the present case were lack of prior vaccination and a potential state-wide outbreak, our patient was treated supportively with antipyretics and antihistamines for his pruritic rash. He was additionally counseled on potential risk factors for acquiring hepatitis A infection and was recommended to follow up with his primary care physician to receive routine age-appropriate vaccinations.

## Conclusions

In a patient presenting with symptoms of an atypical viral syndrome, such as rash and arthralgias as in the presented case, acquiring further history of recent gastrointestinal symptoms or recent exposures can lead to a timely diagnosis. Diligent history-taking can prevent prolonged hospital admission and avoid the financial costs of an extensive medical workup and therapeutic trials, as experienced in this case. This unusual case of hepatitis A, as well as the recent epidemiologic changes of HAV, demonstrates the importance of including hepatitis A infection on a differential diagnosis in patients presenting with a seemingly non-specific viral illness. Furthermore, if hepatitis A virus is included on the differential, it may be prudent to consider retesting for anti-HAV antibody titers if initial titers were negative in patients lacking a unifying diagnosis.
